# The Role of Emotion in Musical Improvisation: An Analysis of Structural Features

**DOI:** 10.1371/journal.pone.0105144

**Published:** 2014-08-21

**Authors:** Malinda J. McPherson, Monica Lopez-Gonzalez, Summer K. Rankin, Charles J. Limb

**Affiliations:** 1 Department of Otolaryngology-Head and Neck Surgery, Johns Hopkins University School of Medicine, Baltimore, Maryland, United States of America; 2 Peabody Conservatory of The Johns Hopkins University, Baltimore, Maryland, United States of America; The University of Chicago, United States of America

## Abstract

One of the primary functions of music is to convey emotion, yet how music accomplishes this task remains unclear. For example, simple correlations between mode (major vs. minor) and emotion (happy vs. sad) do not adequately explain the enormous range, subtlety or complexity of musically induced emotions. In this study, we examined the structural features of unconstrained musical improvisations generated by jazz pianists in response to emotional cues. We hypothesized that musicians would not utilize any universal rules to convey emotions, but would instead combine heterogeneous musical elements together in order to depict positive and negative emotions. Our findings demonstrate a lack of simple correspondence between emotions and musical features of spontaneous musical improvisation. While improvisations in response to positive emotional cues were more likely to be in major keys, have faster tempos, faster key press velocities and more staccato notes when compared to negative improvisations, there was a wide distribution for each emotion with components that directly violated these primary associations. The finding that musicians often combine disparate features together in order to convey emotion during improvisation suggests that structural diversity may be an essential feature of the ability of music to express a wide range of emotion.

## Introduction

Music has been described as the ‘language of emotions’ [1], [2], yet how specific features of music are able to both express and elicit emotions remains poorly understood. While each feature of music (e.g. key, mode, tempo, etc.) contributes to the ability of music to convey emotion, no single element sufficiently accounts for the vast emotional range of music. Complicating this issue is the fact that emotional experiences can often defy simple definition or specification because of their subjective nature and varying intensity.

Thus far, no unified model exists that clearly defines the relationship between musical structure and emotion [1]. Several efforts have focused on identifying a set of universal basic emotions that are expressed through music, generally including happiness, sadness, fear, anger and surprise [3], [4]. Other research has proposed tension and relaxation within music as the basis for a music-specific model of emotion [5], [6]. One of the most commonly used and broadly applicable models defines emotions using the parameters of valence (pleasant vs. unpleasant) and arousal (intensity) [7].

The bulk of knowledge about the relationship between music and emotions comes from studies that examine the perception, rather than the production, of music [1]. Many studies have examined one of the most basic emotional distinctions, that of happiness vs. sadness (sometimes referred to as ‘positive’ vs. ‘negative’), and have found that there are tonal, rhythmic and articulatory differences between ‘happy’ and ‘sad’ emotions. For example, a general correlation exists between perception of happiness and major keys, and sadness and minor keys [8]–[13]. It has also been claimed that specific keys may better express different emotional and aesthetic qualities [14], [15]. The pitch range of compositions has been shown to have direct effects on emotion perception, for example, lower pitches are generally perceived as sadder than higher pitches [16], [17], although very high pitches can be associated with extreme sadness or grief [18], [19]. Additionally, tempo and volume generally increase during happy music and decrease during sad music [9], [10], [20]–[24]. There are also articulation differences between happy and sad compositions; staccato articulations (short notes separated by silence) are generally perceived as happier than legato articulations (no silence between notes and smooth note transitions [20], [25].

Studies focusing on the perception of emotion in music alone, however, have limitations that include the potential biases associated with the selection of music chosen by the experimenter and the difficulty of standardizing subjective reports of emotion. More importantly, these approaches minimize the crucial role played by the composer and performer in conveying musical emotion. In over a century of empirical research about the relationship between emotion and music, very few studies have specifically addressed the production of emotional instrumental music by providing musicians with explicit emotional cues and then analyzing their musical output in order to see how musicians accomplish emotionally-motivated musical tasks [20], [23], [26]–[29]. These previous production studies were extremely important in indicating the enormous complexity of the relationship between emotion and musical production, yet these studies included several experimental constraints that we attempted to address in the current study. Most of these production studies [20], [23], [27]–[30] required musicians to alter pre-determined melodies or rhythms to express a specific emotion. Because of this, their analysis was limited to tempo, articulation, volume and timbre, leaving out such features as key and note range (among others) and also biased the musicians by providing them with an essentially arbitrary template upon which to base their responses. In one of the more recent of these studies [26], pianists were asked to express emotions using improvisations on a single note. This also limited the musicians’ means of conveying each emotion to modulations in volume, tempo and articulation. Consequently, these experimental paradigms suffered from being musically impoverished and somewhat unnatural.

A more subtle but equally important potential confounding element within these previous experiments was the use of verbal or language-based cues to direct the musicians’ performances. There is evidence that language can influence people’s perception of emotional stimuli, and it is possible that linguistic labels for emotions could influence musicians to depict them in stereotypic fashion [31], [32]. Such labels may poorly represent the often transient and subjective nature of emotional content in music, where multiple emotions can be implied by a single musical passage or through a single musical feature [33], [34].

Here we present the first ecologically valid examination of the production of novel emotional music. In this study, we asked professional jazz pianists to improvise short musical pieces (1 min) based on visually presented emotional cues (photographs and cartoons) without any other musical constraints. By using improvisation and visual emotional cues, we sought to develop a more natural account of musical elements that correspond to positive and negative emotional categories. We hypothesized that a broad range of musical features would characterize improvisations to positive and negative emotional targets, rather than a simplistic (driven by one or two key elements) or predictable relationship between emotional target and musical structure. In order to address this hypothesis, we developed novel visual-emotional cues for this study, and assessed the emotional valence of each cue through behavioral testing. We also tested whether naïve listeners perceived any emotional differences between the musical improvisations created by the musicians during the study. Our results demonstrate that musicians employ a diverse range of musical approaches to convey specific emotions in response to emotional cues during unconstrained improvisation. Thus, we argue that musical representations of emotions cannot be sufficiently explained by simplistic correlations (e.g. minor key = sad, major key = happy) between musical features and target emotions. Instead, a broader approach to the diversity of factors that impact emotion in music is crucial to understanding the remarkable ability of music to provide a vast range of deeply emotional experiences.

## Methods

### Stimuli Testing

#### Subjects

Twenty volunteers, 11 males and 9 females (mean age = 32±17 s.d. years, minimum age = 18 years), from the Johns Hopkins University community participated in the stimuli testing. Informed consent was obtained in writing for all subjects and they did not receive monetary compensation for participating. All experimental procedures were approved by the Johns Hopkins University School of Medicine Institutional Review Board.

#### Procedure

**Figure 1:**
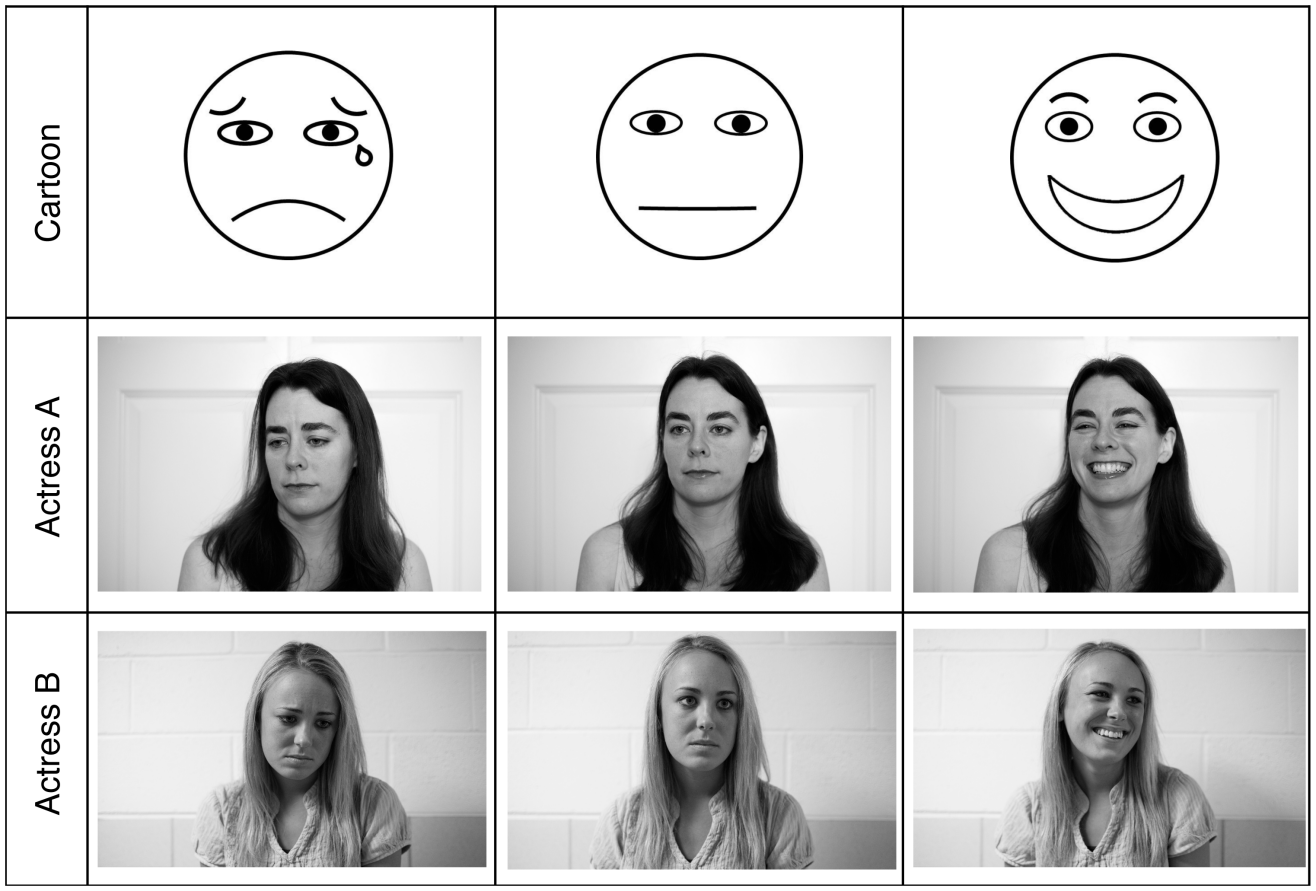
Photographs and Cartoons Used as Visual Stimuli. Cartoon faces representing each of the three emotions were created using Microsoft PowerPoint. The photographs were shot indoors in black and white with a 50 mm lens at f16 using a Nikon D700 digital SLR camera.

**Figure 2:**
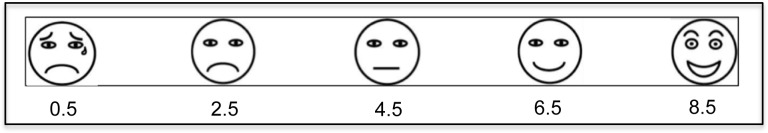
Visual Analog Scale (VAS) with nine point coding rubric below.

We developed a set of cartoons and photographs that represented three basic emotional categories (Positive, Ambiguous and Negative). The actresses pictured in this manuscript have both given written informed consent (as outlined in the PLOS consent form) to publish their photographs. The stimuli consisted of 1 min movies showing either a photograph or cartoon (Figure 1). Each stimulus contained a green dot denoting the beginning, followed by 10 s of a blank screen, followed by 50 s of the emotional cue, and a red dot to denote the end. For the purposes of this study, we used James Russell’s Circumplex model of emotion to define “Ambiguous” as a neither positive nor negative rating in valence and arousal [7], [17], [35]. Subjects were asked to rate the emotion they perceived in each stimulus by marking an ‘X’ on an emoticon Visual Analog Scale (VAS) (Figure 2). The order of the stimuli was randomized for each subject. Subjects were only allowed to view and respond to each image once, and were given an unlimited time to respond.

### Piano Performance

#### Subjects

Fourteen healthy, normal hearing male (11) and female (3) musicians (mean age = 42±15 s.d. years) were recruited from the Peabody Institute of The Johns Hopkins University community. All were full-time jazz piano students or professional musicians who had at least five years of professional jazz piano performance experience (mean years of professional experience = 21±12 s.d., mean years playing piano = 31±14 s.d.). Informed consent was obtained in writing for all subjects, and all subjects received compensation. All experimental procedures were approved by the Johns Hopkins University School of Medicine Institutional Review Board.

#### Procedure

The pianists were seated at a 73-key weighted Korg SV-1 piano keyboard, routed through two Mackie MR5mk2 studio reference monitor speakers. Sound levels were kept constant through the entire session and between subjects. All stimuli (photographs and cartoons) were presented on a 21.5 in iMac (OS X 10.6.8) using Max6 (Cycling 74, Walnut, CA). MIDI (Musical Instrument Digital Interface) information from the piano keyboard was recorded using GarageBand (Apple Inc., Cupertino, CA). The data were analyzed in MATLAB (MathWorks, Inc., Natick, MA) using MIDI Toolbox [36] and custom scripts. The authors verified the mode and key analyses by visual inspection of the scores.

Half of the pianists saw the photographs of Actress A, and the other half saw only the photographs of Actress B. Control blocks (blank white screens) were intermixed with the stimuli. The blank screen control blocks contained a green dot denoting the beginning and a red dot denoting the end. The order of the stimuli was randomized for each subject. Pianists were instructed to look at the monitor, and not their hands, for the duration of the experiment.

The experiment was divided into four parts. During the first part, pianists were familiarized with the six emotional stimuli (three cartoons and a subset of three pictures) by viewing each full video clip. During the second portion of the experiment, pianists viewed each stimulus again, and were instructed to improvise a novel composition using both hands and the full range of the keyboard. During the third part of the experiment, pianists were asked to view the same stimuli and improvise a monophonic piece (one note at a time) using their right hand. They were restricted to using a 2.5 octave range (C3 to B-flat 5). The fourth part of the experiment was an exact repetition of part two. Pianists were asked to improvise compositions that matched the emotions expressed in the images (See File S1 for full instructions). For the blank screen control conditions, which were intended to have no emotional connotations, pianists were instructed to improvise freely. Examples of the stimuli and responses are available online (https://www.youtube.com/user/LimbMusicLab).

### Listening Survey

#### Subjects

Twenty healthy subjects (mean age = 24±5 s.d. years), including ten musicians (4 males, 6 females, with mean years of musical training = 10.7±1.82 s.d.) and ten non-musicians (5 males and 5 females), were recruited from Johns Hopkins University and the greater Baltimore area. Informed consent was obtained in writing for all subjects and they did not receive monetary compensation for participating. All experimental procedures were approved by the Johns Hopkins University School of Medicine Institutional Review Board.

#### Procedure

Each subject heard a random sample of improvisations from the piano performance portion of the study. This random sample included four improvisations from each emotional category (Positive, Negative and Ambiguous), with two one-handed and two two-handed improvisations for each emotion. There were a total of ten randomizations – one non-musician and one musician heard each randomization. The subjects listened to the last 50 s of each improvisation through headphones.

Subjects were asked to rate the emotion that they believed the improvisation was expressing by marking an emoticon Visual Analog Scale (Figure 2) with an ‘X’. Subjects were allowed to listen to each improvisation once, and were given an unlimited time to respond.

## Results

The stimulus testing was conducted to confirm that our visual stimuli were appropriate emotional cues for the piano performance testing. Results were coded using a nine point scale, with 0 = the most negative, 4.5 = ambiguous, 9 = the most positive. Due to the orthogonal nature of the data, a two-way ANOVA on the ratings with within-subject factors Emotion (Negative, Ambiguous, Positive) by Type (Cartoon, Actress A, Actress B) was calculated to compare the ratings between conditions [37]. Significant main effects of Emotion, [*F*(1, 2) = 564.65, *p*<.001] and Type, [*F*(1,2) = 15.54, *p*<.001] were observed and their interaction was significant [*F*(1, 4) = 26.72, *p*<.001].

Mean ratings for the Negative stimuli: Cartoon = 0.5±0.94 (s.d.); Actress A = 3.7±0.92 (s.d.); Actress B = 2.85±1.37 (s.d.). Mean ratings for the Ambiguous stimuli: Cartoon = 4.43±0.59 (s.d.); Actress A = 4.75±0.75 (s.d.); Actress B = 3.95±.74 (s.d.). Mean ratings for the Positive Stimuli: Cartoon = 8.35±0.33 (s.d.); Actress A = 7.65±0.80 (s.d.); Actress B = 7.2±0.99 (s.d.).

### Piano Performance

The following are multiple analyses that were performed on the data from the improvisations. We analyzed the final 50 s of each trial (during the first 10 s of each trial the pianists were presented with a blank screen). For the measures Note Density, Note Range and Key Press Velocity, we ran separate one-way ANOVAs with factor Trial (Trial 1 and Trial Three, the two-handed trial) to test for an effect of trial order. Because no significant (*p*>.05) effect of Trial (trial order) was found, the two-handed trials were analyzed together. For all analyses except key and note transitions (overlaps and silences) analyses were run separately for one and two-handed improvisations.

#### Note Density

**Figure 3:**
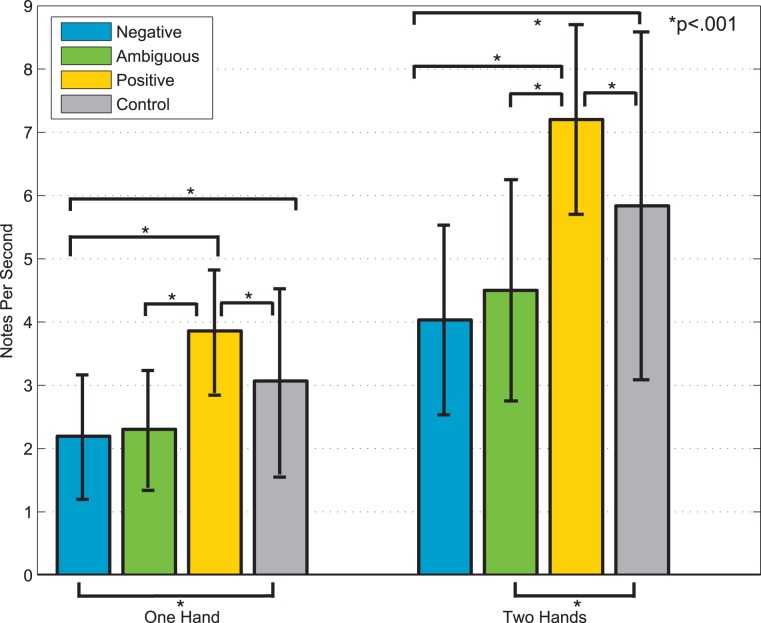
Average note density of one-handed and two-handed improvisations.

Note density is a measure of average notes per second (Figure 3). Note density can be used as a strong indicator of tempo in monophonic improvisations and a weaker indicator of tempo in polyphonic improvisations, as chords or ornaments such as trills can increase the number of notes per second even if the absolute tempo does not increase. We calculated a two-way ANOVA on note density with within-subject factors Emotion (Negative, Ambiguous, Positive) and Type (Cartoon, Actress). Because no significant (*p>*.05) effects of Type were found, we collapsed the data by Type, and the ANOVA was rerun as a one-way ANOVA with the within-subject factor Emotion (Negative, Ambiguous, Positive). Tukey’s honestly significant difference criterion was used for post-hoc comparisons. A main effect of Emotion was found for two-handed [*F*(1,3) = 99.65, *p*<.001] and one-handed trials [F(1,3) = 53.28, *p*<.001]. For both one- and two-handed trials, a significant difference between Positive and Negative conditions was found (*p*<.001). Note density was significantly different (*p*<.001) between Ambiguous and Positive trials, Positive and Negative trials, and between all emotions and the Control. There was no statistically significant (*p*>.05) difference between the note densities of Ambiguous and Negative trials. Higher note densities were used to express Positive emotions, and lower note densities were used to express Negative and Ambiguous emotions.

#### Duration Distribution

**Figure 4:**
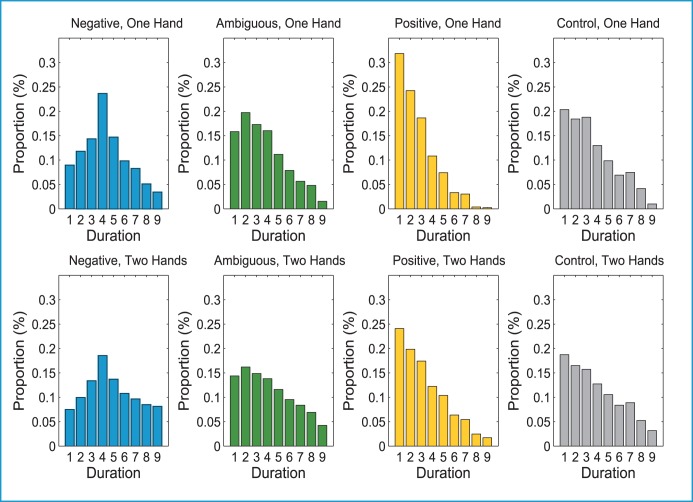
Distributions of note durations for one-handed and two-handed improvisations.

The duration distribution function of the MIDI Toolbox returns the percentage of notes that fall into nine different logarithmically organized bins (note length categories). Length categories are defined as a unit of beats. We set our MIDI tempo so that 1 beat = .5 s (quarter note = 120 Beats Per Minute (BPM)). Therefore, bin 1 = 1/8 s, bin 3 = ¼ s, bin 5 = ½ s, bin 7 = 1 s, and bin 9 = 2 s. The relationship between bin 1 and bin 9 is proportional to the relationship between a sixteenth note and a whole note. Two-sample Kolmogorov-Smirnov tests showed that there were statistical differences (*p*<.05) between corresponding bins of the distributions for Negative, Positive and Ambiguous for both one- and two-handed improvisations. Ambiguous and Control conditions were not statistically different in either condition (Figure 4).

During the two-handed control condition, 63.75% of the notes were less than 1 s in duration, which was similar to the 57.5% of notes that were less than 1 s during Ambiguous trials. During Positive improvisations, 24.8% of the notes were ⅛ of a second or less, and 73.5% of the notes were less than 1 s. When musicians improvised to the Negative emotion, only 46.94% of the notes were less than 1 s in length.

#### Key Press Velocity

**Figure 5:**
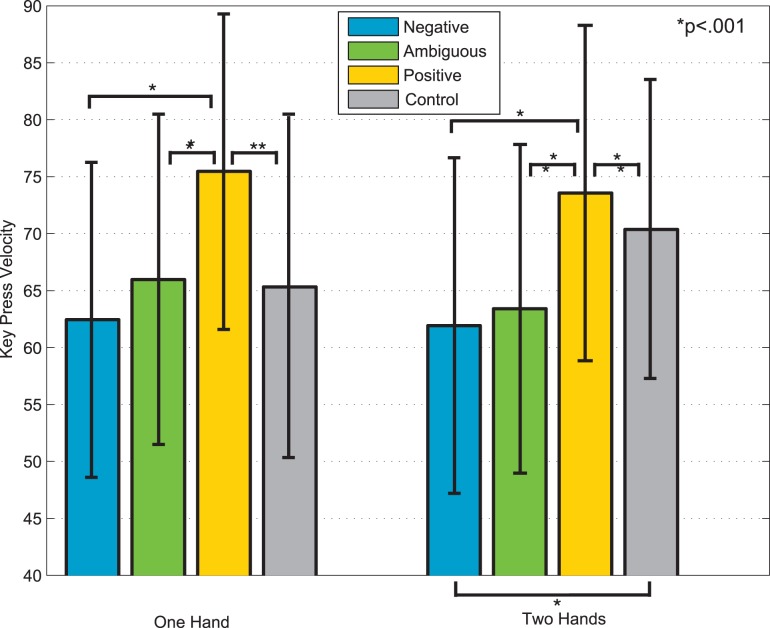
Average key press velocity for one-handed and two-handed improvisations.

Velocity is the measurement of how quickly a key was depressed, and is linearly related to sound pressure level (SPL) [36], [38]. Our results show that Positive improvisations tended to be louder than Negative or Ambiguous improvisations (Figure 5). We calculated a two-way ANOVA on mean key press velocities with factors Emotion (Negative, Ambiguous, Positive) and Type (Cartoon, Actress). Because no significant (*p*>.05) effects of Type were found, we collapsed the data by Type, and the ANOVA was rerun as a one-way ANOVA with the within-subject factor Emotion (Negative, Ambiguous, Positive). Tukey’s honestly significant difference criterion was used for post-hoc comparisons. A main effect of Emotion was found for two-handed [*F*(1,3) = 45.69, *p*<.001] and one-handed trials [*F*(1,3) = 23.51, *p*<.001]. For both and one- and two-handed trials, Positive key press velocities were significantly greater (*p*<.001) than Negative, Ambiguous and Control key press velocities. The difference between the Control improvisations and Negative improvisations for two hands was also significant (*p*<.001).

#### Note Transitions: Overlaps and Silences

**Figure 6:**
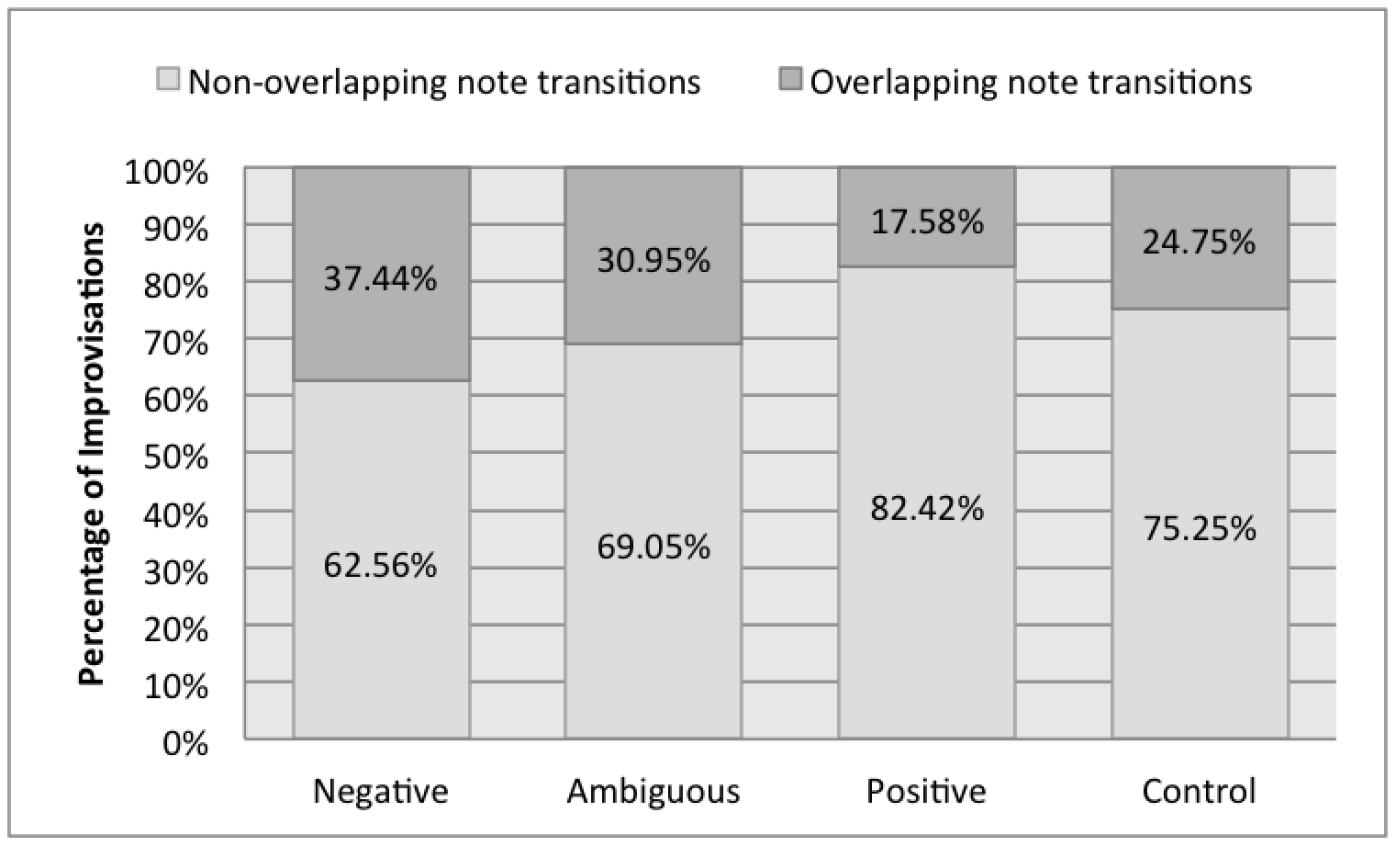
Overlapping and non-overlapping note transitions during one-handed improvisations.

Though the pianists were instructed to make their one-handed improvisations completely monophonic, we found that their notes overlapped by fractions of a second when they attempted to create the effect of legato. Conversely, when trying to create the effect of non-legato or staccato, there were silences between the notes. We examined the proportion of overlapping and non-overlapping note transitions for each emotion. There were over twice as many overlapping note transitions in Negative improvisations compared to Positive improvisations (Figure 6).

#### Note Range, Maximum and Minimum

**Figure 7:**
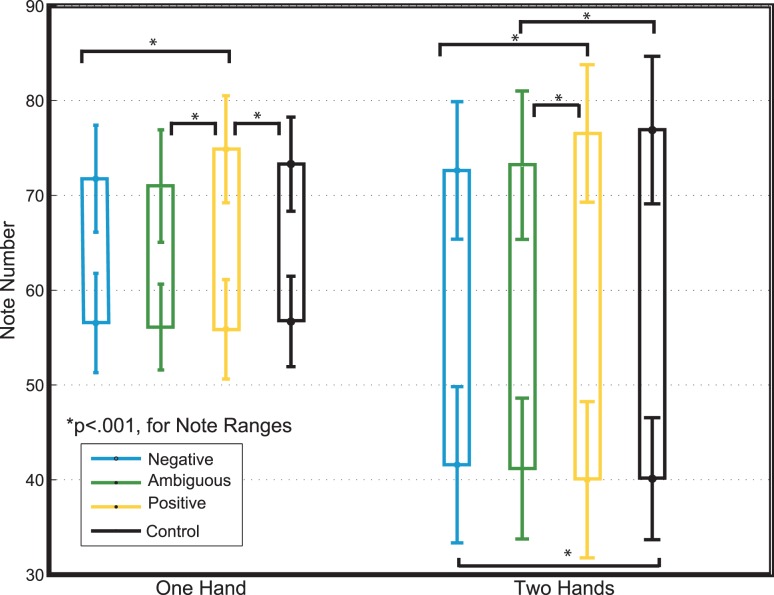
Significant differences between note maximums but not note minimums in both one-handed and two-handed improvisations between all emotions.

We calculated two-way ANOVAs on the note (pitch) minimum, note maximum, and note range (difference between highest and lowest notes during improvisation) using within-subject factors Emotion (Negative, Ambiguous, Positive) and Type (Cartoon, Actress). Because no significant (*p*>.05) effects of Type were found for note range, we collapsed the data by Type, and the ANOVA was rerun as a one-way ANOVA using the within-subject factor Emotion (Negative, Ambiguous, Positive). Tukey’s honestly significant difference criterion was used for post-hoc comparisons. A main effect of Emotion was found for two-handed [*F*(1,3) = 30.69, p<.001] and one-handed trials [*F*(1,3) = 18.34, *p*<.001] (Figure 7). For both two- and one-handed trials, a significant difference between Positive and Negative conditions was found (*p*<.001). This was primarily accounted for by differences in the note maxima (*p*<.001), not the note minima. There was no statistically significant difference (*p*>.05) in note minima between any of the emotions or the control for one handed improvisations, and for two-handed improvisations, only Positive and Negative improvisations were significantly (*p*<.05) different. There were no significant (*p*>.05) differences between Ambiguous and Negative note ranges. Our results indicate that a wider range in pitch is more highly correlated with the Positive condition, but this is mainly accounted for by differences in note maxima between emotions, not note minima. During improvisation, jazz musicians use higher tones to show happiness, but do not, conversely, use lower tones to show negative emotions.

#### Mode

Key (tonal center) and mode were calculated using the Krumhansl & Schmuckler (K–S) key-finding algorithm, which uses the pitch class distribution of a piece (weighted according to duration) to return a key profile for the piece [36], [39]–[41]. We used the K–S key finding algorithm to find the best fit for each entire 50 s improvisation.

**Figure 8:**
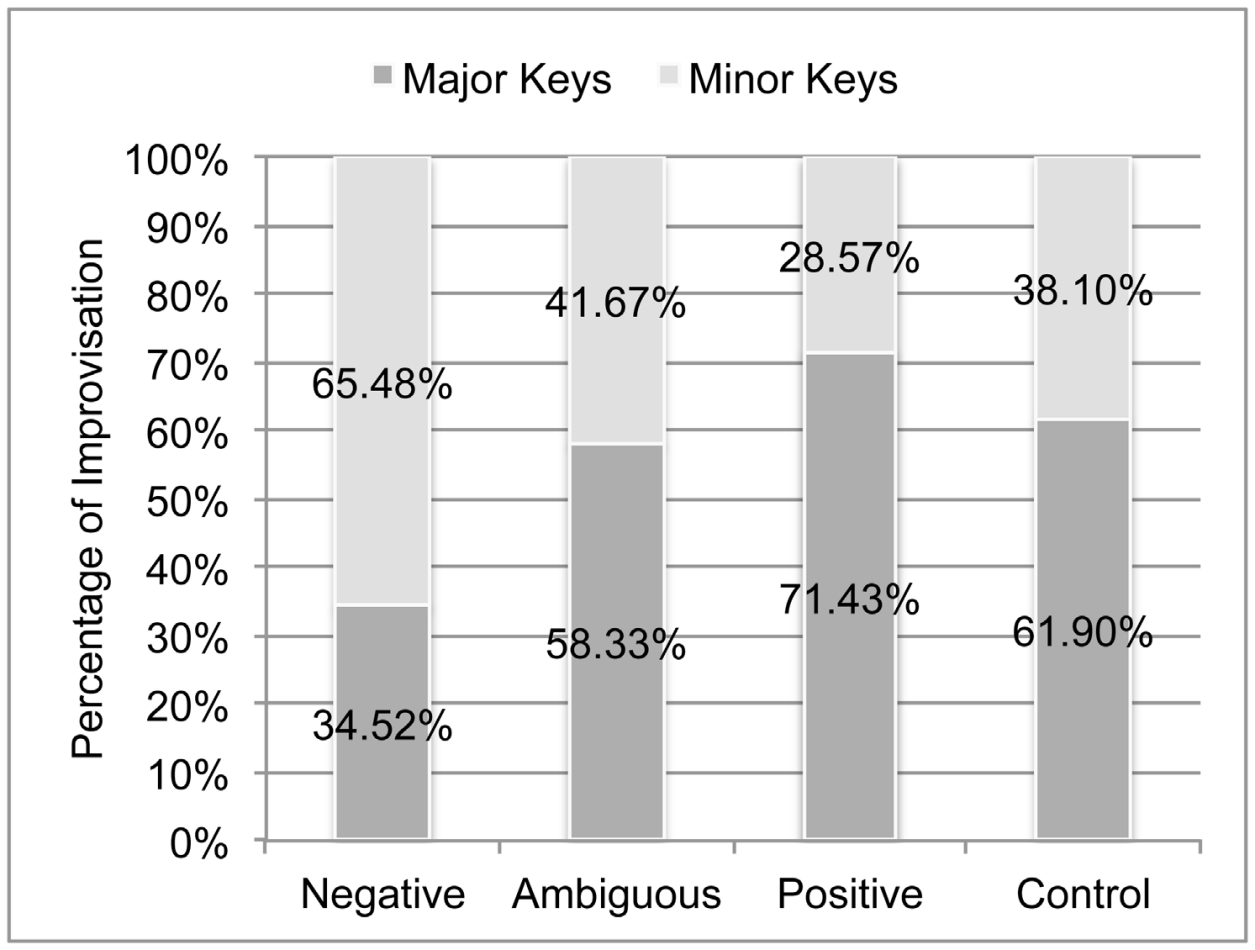
Differences in proportion of major to minor keys in Positive, Ambiguous and Negative improvisations.

There was a large amount of variation within each Emotion category (combined across one-handed and two-handed improvisations); 34.52% of the Negative improvisations were in a major key, and conversely, 28.57% of the Positive improvisations were in a minor key. The Ambiguous and Control improvisations showed almost identical proportions of major (58.33% and 61.9%, respectively) to minor (Figure 8). We conducted a follow-up analysis to determine whether there were any velocity, range or note density differences between major and minor improvisations within any given emotional category or the control. A two-tailed independent t-test was used to compare the ranges, velocities and note densities of major to minor improvisations within each Emotion. There were no significant (*p*>.01) differences between any note densities, key press velocities or ranges of major vs. minor improvisations within any emotion or the Control. This result shows that there was not a significant interaction between mode and other musical variables.

#### Key

**Figure 9:**
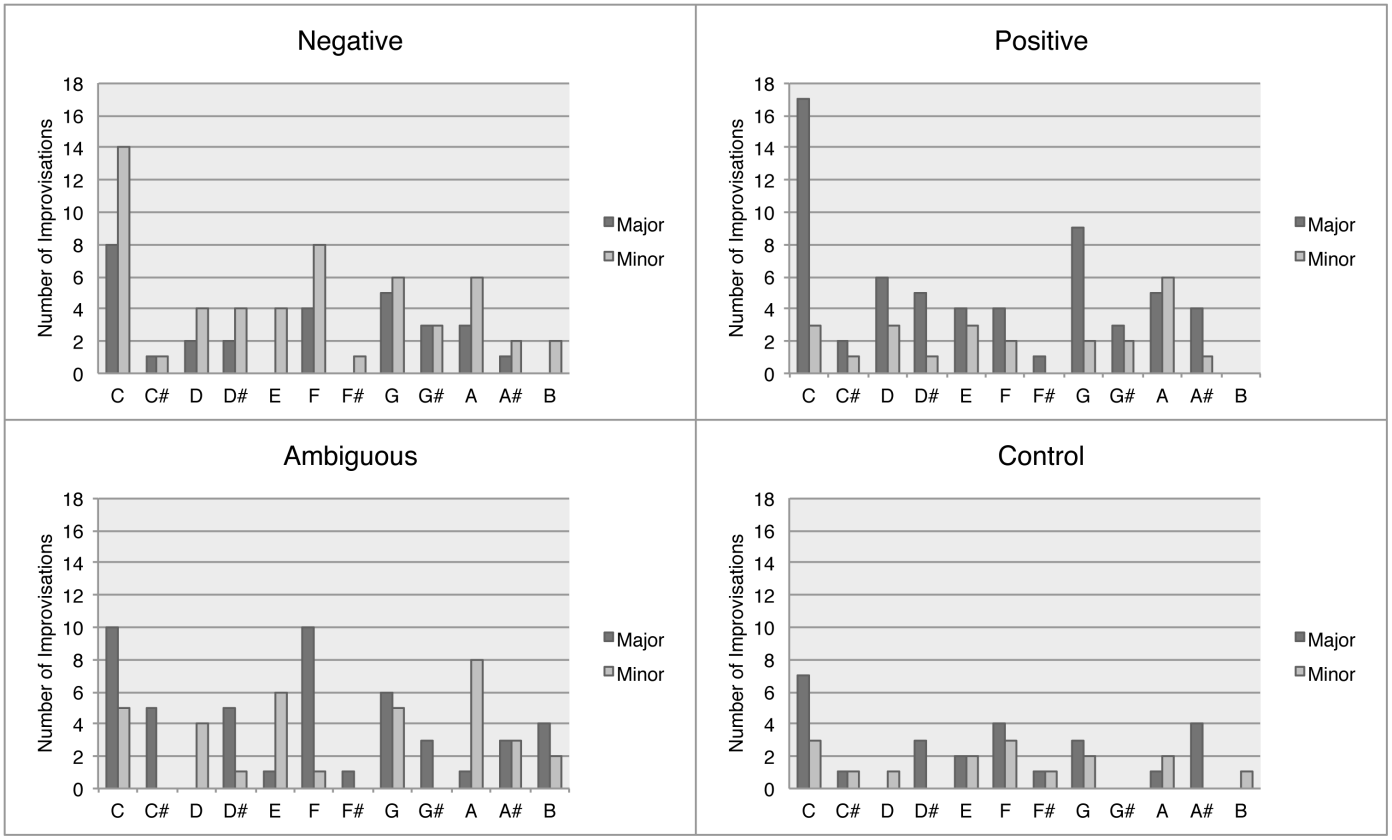
Histograms of keys used during improvisations, separated by emotion.

With respect to key, the overall tendency was to use A, C, F and G, each in both major and minor, and to use keys with sharps for positive improvisations and keys with flats for negative improvisations (Figure 9).

### Listening Survey

The listening survey showed that subjects perceived a difference between Positive and Negative improvisations and between Positive and Ambiguous improvisations, however they perceived Ambiguous and Negative improvisations as similar. We found that musical experience did not influence subjects’ emotional evaluations. Furthermore, improvisations made in response to cartoon and photographs were equally emotionally convincing, and emotional evaluations were unaffected by whether the improvisations were monophonic or polyphonic (performed with one hand or two hands). At least within the realm of piano performance, single melodic lines appear to be as emotionally convincing as polyphonic performances – the musical features present in a monophonic composition appear to be sufficient to effectively convey an emotion.

Results were coded using a nine point scale rubric, with 0 = the most Negative, 4.5 = Ambiguous, 9 = the most positive. We calculated a four-way ANOVA on the ratings with within-subject factors Musical Experience (musician, non-musicians), Emotion (Negative, Ambiguous, Positive), Type (Cartoon, Actress) and Hands (one-handed and two-handed improvisations). Because no significant effects (*p*>.05) of Musical Experience, Type, and Hands were found, the ANOVA was rerun as a one-way ANOVA with the within-subject factor Emotion (Negative, Ambiguous, Positive). A significant main effect of Emotion [*F*(1, 2) = 45.12, *p*<.001] was observed. The ratings of Positive improvisations were significantly greater (*p*<.001) than ratings for Negative and Ambiguous improvisations. There was no statistical difference (*p*>.05) between Negative and Ambiguous ratings. The mean ratings: Negative improvisations = 3.46±1.61 (s.d.); Ambiguous improvisations = 3.51±1.71 (s.d.); Positive Improvisations = 5.63±1.63 (s.d.). The range of responses for Negative improvisations was 7 points, 6.5 points for Ambiguous improvisations and 7.5 for Positive improvisations (i.e. some Negative improvisations were rated as very positive, and vice versa).

## Discussion

Music is viewed as an effective means of expressing emotions, yet there is a large amount of variability in how music can express emotions. Unlike language, where words describing emotions have distinct, agreed upon meanings, the emotional content of music is transient and non-discrete. Multiple emotions can be evoked within a single musical passage. It has been posited that the power of music derives precisely from this fluidity [33]. While this indeterminacy would make propositional language unfeasible, ambiguity of meaning makes music more powerful by allowing each person to ascribe their own meaning to pieces of music [33]. The fact that a broad range of musical features can express a given emotion supports the idea that music can express the same emotion in different ways. Individual features of music can be more strongly associated with specific emotional valences, but independently, a single musical feature cannot explain the musical expression of emotions.

The objective of this study was to explore the range of musical features that jazz pianists use to express emotions while improvising. Our experimental design allowed us to examine emotional music performance in an artistically and ecologically valid setting, and we found that the emotional cue and subsequent emotional intent of the performers greatly influenced all measured musical elements of their performance. Statistical differences were observed in every musical measure between Positive and Negative improvisations. The differences between Ambiguous and Negative improvisations were not as pronounced. There were no statistical differences between Ambiguous and Negative improvisation note densities, ranges, or velocities. Percent of Legato/Staccato notes only differed by approximately 7% between Ambiguous and Negative improvisations. However, almost twice as many Ambiguous improvisations were major compared to Negative improvisations, and the duration distributions for Ambiguous and Negative improvisations were significantly different. Further statistical tests revealed that note density, key press velocity, and note range varied independently of mode - improvisations that did *not* conform to the conventional mode (e.g., a Positive piece played in a minor key), did not show exaggerated emotional effects across other parameters (e.g., faster tempo, higher velocity, more staccatos). Performers did not “compensate” for their choice of mode using other musical measures.

The musical similarities between Ambiguous and Negative improvisations are particularly striking given that the emotional ratings for the Ambiguous and Negative stimuli were significantly different. In an informal post-study survey, four pianists independently stated that the Ambiguous stimuli made them feel “anticipation”, which some claimed they had expressed through a lower range, monotone textures, and dissonance. Others commented that the Ambiguous faces were more difficult to musically ‘match’ because they were simply “not emotional”. One pianist stated that the “guy with the line mouth” (referring to the Ambiguous cartoon) “didn’t inspire anything”. These statements provide an indication of why Ambiguous and Negative improvisations may have shared certain characteristics. Pianists’ anticipation, uncertainty or even lack of emotional response to the Ambiguous stimuli contributed to their use of narrow range, slow tempo, and low volume. Ambiguity is, by definition, open to many different interpretations. Perhaps cueing the pianists to improvise something ambiguous caused them to be uncertain of what to do.

It is important to note that, even when statistically similar to Negative improvisations, the mean values for all Ambiguous improvisation measures (other than the mean one-handed note maximum) fell between the means for Negative and Positive improvisations. Furthermore, pianists’ choice of mode during Ambiguous improvisations was almost at chance level (41.67% minor, 58.34% major), compared to Negative improvisations, where 65.48% of improvisations were in minor keys. Ambiguous trials were more similar to Positive trials than Negative trials with respect to mode. This may be further indication that gross similarities between certain musical features of Ambiguous and Negative improvisations are not necessarily an indication that the pianists’ Ambiguous improvisations were tending towards expressing negative emotions. The pianists may have simply been less effortful and expressive during Ambiguous trials. Using less physical effort could have resulted in lower volume, smaller range and fewer notes, but would have had no effect on choice of mode.

Regardless of what emotion they were trying to convey, the pianists used a wide range of musical features. This may be attributed to the fact that the pianists were spontaneously producing emotionally guided music rather than composing (pre-planning what they would perform). In the post-study interview, all fourteen subjects independently stated that they were using minor keys during Negative improvisation trials and major keys during Positive improvisation trials. These responses are consistent with the Western Classical music convention that major keys are happy and minor keys are sad [13], [42]. Upon quantitative analysis, we discovered that this was not fully the case. While a majority of Negative improvisations were in minor keys and the majority of Positive improvisations were in major keys, a large percentage of Negative improvisations were in major (34.52%) and Positive improvisations were in minor keys (28.57%). Therefore, during approximately ⅓ of the Positive or Negative improvisations, pianist’s behavior did not match their verbal reports of what they thought they did during the experiment. If pianists had been given more time to plan their improvisations (taken time to write out compositions, for example), their use of musical features may have been less varied, as they might have more closely adhered to specific Western Classical music conventions for expressing emotions.

We also believe that our use of visual cues impacted the range of musical features used within each emotional category. We decided to use visual cues in order to eliminate all external verbal labels of emotion from our study, as linguistic labels can bias emotion perception and report [31], [32]. We observed a significant main effect of Type (Actress A, Actress B, Cartoon) as well as Emotion (Negative, Ambiguous, Positive) on the emotional ratings of stimuli in the listening survey. Subjects perceived the two Actresses and Cartoons as portraying slightly different emotional valences (though there was still a main effect of Emotion). There was not a similar effect of Type on musical improvisations. If the musicians were trying to precisely ‘match’ the emotion represented in the cues, it is likely that there would have been differences between the improvisations in response to each actress and cartoon. This did not occur. Instead, it seems as if the pianists used the images as more general, rather than specific, emotional cues, resulting in a wider range of musical expression.

Furthermore, the pianists were instructed to make their improvisation as a whole express the emotion they perceived in the stimuli. Improvisation is the unfolding of multiple events over time, and emotion expressed in music is an emergent property of the entire piece of music. This task left significant room for the pianists to musically and emotionally fluctuate, as long as the overall emotion expressed matches that of the stimuli. Musical emotions may not have a high level of specificity and regularity. While faces can convey a single emotion (‘Happy’, ‘Sad’, etc.), or compound emotions such as ‘Happily surprised’ or ‘Fearfully angry’ [43], perhaps music primarily expresses multiple, intermixed emotions rather than isolated emotions. This could help account for music’s universal appeal, and the ability for cross-cultural recognition of musical emotions [44]. It appears that there are general methods to express certain emotional categories, however a large amount of freedom exists within those general approaches.

The wide distribution of musical features likely accounts for the large range of responses observed in the listening survey. The listening survey revealed that subjects were generally able to discern Positive improvisations from Ambiguous and Negative improvisations, but that the difference between Ambiguous and Negative improvisations was not as clear. Previous studies have found that mode is a particularly strong predictor of emotional perception [10], [45], yet the mode differences between Ambiguous and Negative improvisations were clearly not sufficient to change people’s ratings of the two different emotional categories. This suggests that features such as tempo and articulation may be more important than features such as key when it comes to making emotional judgments.

Our findings demonstrate that a strict correspondence between emotions and musical features (i.e., Positive-major, Negative-minor) does not explain the diversity of musical expression of emotion. Instead, our results support the hypothesis that there is a high amount of musical variety within each emotional category. Rather than using a simple set of features to express emotions, the pianists used many permutations of features in order to express different emotions. While this high degree of structural variation in music may be particularly pronounced during spontaneous improvisation in comparison to other forms of musical expression, we believe that this enormous variety is directly related to the broad capacity of music to provide compelling, vivid and fluid emotional experiences that are often difficult to describe.

## Supporting Information

File S1 **Instructions for Pianists.**
(EPS)

File S2 **Public Date.**
(ZIP)
